# Hydroxytyrosol Promotes the Mitochondrial Function through Activating Mitophagy

**DOI:** 10.3390/antiox11050893

**Published:** 2022-04-30

**Authors:** Yanzou Dong, Manhan Yu, Youlin Wu, Tian Xia, Ling Wang, Kai Song, Chunxiao Zhang, Kangle Lu, Samad Rahimnejad

**Affiliations:** 1 Key Laboratory for Feed Quality Testing and Safety, Fisheries College, Jimei University, Xiamen 361021, China; yan_zoudong@163.com (Y.D.); 201911710013@jmu.edu.cn (M.Y.); 202011908013@jmu.edu.cn (T.X.); lingwang@jmu.edu.cn (L.W.); songkai@jmu.edu.cn (K.S.); cxzhang@jmu.edu.cn (C.Z.); 2 Key Laboratory of Swine Nutrition and Feed Science of Fujian Province, Fujian Aonong Biological Science and Technology Group Co., Ltd., Zhangzhou 363000, China; wuyoulin@aonong.com.cn; 3 South Bohemian Research Center of Aquaculture and Biodiversity of Hydrocenoses, Faculty of Fisheries and Protection of Waters, University of South Bohemia in Ceske Budejovice, Zatisi 728/II, 389 25 Vodnany, Czech Republic; srahimnejad@frov.jcu.cz

**Keywords:** hydroxytyrosol, mitochondrion, fat deposition, non-alcoholic fatty liver disease, fish model

## Abstract

Emerging evidence suggests that mitochondrial dysfunction mediates the pathogenesis for non-alcoholic fatty liver disease (NAFLD). Hydroxytyrosol (HT) is a key component of extra virgin olive oil which can exert beneficial effects on NAFLD through modulating mitochondria. However, the mechanism of the impacts of HT still remains elusive. Thus, an in vivo and a series of in vitro experiments were carried out to examine the impacts of hydroxytyrosol (HT) on lipid metabolism and mitochondrial function in fish. For the in vivo experiment, two diets were produced to contain 10% and 16% fat as normal-fat and high-fat diets (NFD and HFD) and two additional diets were prepared by supplementing 200 mg/kg of HT to the NFD and HFD. The test diets were fed to triplicate groups of spotted seabass (*Lateolabrax maculatus*) juveniles for 8 weeks. The results showed that feeding HFD leads to increased fat deposition in the liver and induces oxidative stress, both of which were ameliorated by HT application. Furthermore, transmission electron microscopy revealed that HFD destroyed mitochondrial cristae and matrix and induced severe hydropic phenotype, while HT administration relieved these alterations. The results of in vitro studies using zebrafish liver cell line (ZFL) showed that HT promotes mitochondrial function and activates PINK1-mediated mitophagy. These beneficial effects of HT disappeared when the cells were treated with cyclosporin A (Csa) as a mitophagy inhibitor. Moreover, the PINK1-mediated mitophagy activation by HT was blocked when compound C (CC) was used as an AMPK inhibitor. In conclusion, our findings demonstrated that HT alleviates fat accumulation, oxidative stress and mitochondrial dysfunction, and its effects are deemed to be mediated via activating mitophagy through the AMPK/PINK1 pathway.

## 1. Introduction

The increasing prevalence of obesity in the general population has drawn much attention across the globe. A recent epidemiological study revealed that the prevalence of overweight/obesity between 1980 and 2013 has increased from 28.8% to 38% in humans [1]. Along with increased obesity, non-alcoholic fatty liver disease (NAFLD) has become a prevalent diagnosis in clinical practices [2]. NAFLD refers to fat accumulation in hepatocytes which is often associated with chronic inflammation and metabolic syndrome [3]. Moreover, it can prime a more increased risk of non-alcoholic steatohepatitis (NASH) and cirrhosis when subsequent metabolic stressors emerge [4].

Mitochondria is the main site of fatty acid oxidation; thus, mitochondrial dysfunction-induced retardation of oxidation is inferred as the direct cause for excessive fat accumulation in liver [5]. Thus, NAFLD is often considered as a mitochondrial disorder. Abnormal hepatic mitochondrial function has been reported in patients suffering from NAFLD [6]. Furthermore, the impaired mitochondrial function can lead to continuous production of ROS resulting in oxidative stress and mtDNA damage [7]. Hence, a vicious cycle is presented due to the increasing lipid peroxidation, mitochondrial dysfunction, and reactive oxygen species (ROS) generation [4]. Moreover, the overproduction of ROS results in inflammation through direct activation of the host inflammatory signaling pathways [5]. Moreover, the emerging evidence suggests that mitochondrial dysfunction mediates the pathogenesis for NAFLD [8].

Based on the above, NAFLD could be treated by preventing or reversing liver injury by reducing fat deposition and ameliorating mitochondrial dysfunction. Hence, pharmacological therapies acting on mitochondria are drawing attention as novel strategies for the prevention of NAFLD. Some natural bioactive compounds are used as potential therapeutic agents for modulating mitochondrial function termed as “mitochondrial nutrients” [9,10]. They beneficially influence mitochondrial function, and have been increasingly used as prophylactic agents in controlling NAFLD in different animal models [6,11,12]. Hydroxytyrosol (HT) is a key component of extra virgin olive oil which exerts many beneficial biological activities such as scavenging free radicals and ameliorating fatty liver and diet-induced obesity [13,14]. Moreover, our previous study indicated that HT attenuated liver damage induced by high-fat diet [15]. These properties are deemed to be partially mediated through modulating mitochondrial function and the respiratory chain complex [16]. However, the molecular mechanism(s) of HT on fat deposition and mitochondrial function still remain(s) to be elucidated.

The processes of lipid synthesis, deposition and degradation have been demonstrated to be highly conserved among species [17,18]. Although rodent models have provided great insight to our understanding of NAFLD, the studies using rodent models are relatively costly. Recently, fish models are being increasingly used for investigating obesity and NALFD due to the convenience and comparatively lower cost of experimentation [19,20], as their digestive organs, adipose tissues and skeletal muscles are physically arranged in a manner similar to human counterparts [21]. Furthermore, our previous studies have clearly pointed out that there are strong analogies between fish and mammals in metabolic diseases caused by high-fat diet at the molecular, physiological, and biochemical levels [12,22,23]. Therefore, the present study was conducted to explore the molecular mechanism(s) of HT effects on fat deposition and mitochondrial function using the fish model.

## 2. Materials and Methods

### 2.1. In Vivo Study

#### 2.1.1. Experimental Diets, Feeding Trial, and Sample Collection

A diet containing 10% fat was used as a normal-fat diet (NFD), and a high-fat diet (HFD) was prepared to contain 16% fat. Two HT-containing diets were produced by supplementing 200 mg/kg HT in NFD and HFD (NFD + HT and HFD + HT diets) (Supporting Table S1).

The feeding trial was conducted at Jimei University. After a 4-week acclimation period, 300 spotted seabass juveniles of similar size (11.2 ± 0.1 g) were randomly distributed into 12 tanks of 200 L capacity in a recirculating aquaculture system (RAS) at a density of 25 fish per tank. Each treatment was randomly assigned to three tanks and the fish were fed with the experimental diets to apparent satiation two times daily (08:30 and 17:00) for 8 weeks. Fish were reared in freshwater under the following conditions: temperature from 25.0 °C to 27.5 °C, dissolved oxygen between 5.2 and 6.0 mg/L, and pH ranging from 7.0 to 7.5. Photoperiod was kept on a natural daylight cycle.

At the end of the feeding trial, all fish were fasted for 24 h and individually weighed. Then, six randomly captured fish per tank were anesthetized with 100 mg/L MS-222, their plasma and liver samples were collected according to our previous study [22] and stored at −80 °C until analysis.

#### 2.1.2. Plasma Biochemical Parameters

Activities of aspartate aminotransferase (AST) and alanine aminotransferase (ALT) were detected using commercial assay kits according to the manufacturer’s instructions (Nanjing JianCheng Bioengineering Institute, Nanjing, China). The fat content of liver was extracted following the Folch’s method [24], and the contents of triacylglycerols (TAG), total cholesterol (TC), and non-esterified free fatty acids (NEFA) in the ethanol dissolved fat were detected using commercial assay kits (Nanjing JianCheng Bioengineering Institute, Nanjing, China).

#### 2.1.3. Oxidative Status

To measure the level of ROS, the liver homogenates were incubated at 37 °C for 30 min with 10 μM DCFH/DA (Beyotime, Shanghai, China). The fluorescence of dichlorofluorescein was measured at the excitation wavelength of 500 nm and the emission wavelength of 525 nm by a microplate reader (Varioskan Lux, Thermo Scientific, Waltham, MA, USA). Superoxide dismutase (SOD) activity and malondialdehyde (MDA) concentration were measured according to our previous study [25]. Protein concentration in the liver homogenate was detected by the bicinchoninic acid assay (BCA) method, using a commercial kit (LABLEAD. Inc., Beijing, China).

#### 2.1.4. Liver Histology

For oil red O staining, the liver samples were dehydrated in sugar solution embedded in OCT-embedding medium. After that, samples were cut into sections of 10 μm with a freezing microtome (CRYOSTAR NX50, Thermo Scientific, Waltham, MA, USA). Then, the sections were stained with oil red O and hematoxylin solutions. Observations were carried out under a light microscope (Leica DM5500B).

For ultrastructural analysis, the liver samples were rapidly cut into 1 mm^3^ sections and fixed in cold 2.5% glutaraldehyde solution. Then, after fixing in 1% OsO_4_ for 2 h, the samples were resin-embedded and 60 nm ultrathin slices were made. The slices were stained with heavy metal. Observation and photograph were conducted using a transmission electron microscope (Hitachi H-7650, Tokyo, Japan) at 80.0 kV.

### 2.2. In Vitro Study

#### 2.2.1. Cell Culture

Zebrafish liver cell line (ZFL) was obtained from the Chinese Zebrafish Resource Center (CZRC). The basic medium used in this study contained 50% L15 medium, 30% DMEM/F12 medium, and 30% DMEM medium, named as LDF medium. The ZFL was cultured in the LDF medium containing the following components: 6% fetal bovine serum, 15 mM HEPES, and 1× Pen Strep solution (Gibco, Grand Island, NY, USA) at 28 °C in a CO_2_ incubator.

Exp. I: There were three groups: the Control, FA, and FA + HT groups. Cells of the Control group were cultured using the LDF medium. Cells of the FA group were cultured in FA medium (LDF medium with 0.25 mM oleic acid and 0.25 mM palmitic acid) for 24 h. Cells of the FA + HT group were cultured in FA medium with 50 μM HT for 24 h.

Exp. II: There were three groups: the FA, FA + HT, and FA + HT + Csa groups. The treatments of the FA and FA + HT groups were the same as in Exp. I. Cells of the FA + HT + Csa group were firstly treated with a mitophagy inhibitor (Cyclosporin A, 5 μM, 4 h) and then cultured in FA medium with 50 μM HT for 24 h.

Exp. III: There were three groups: the FA, FA + HT, and FA + HT + CC groups. The treatments of the FA and FA + HT groups were the same as Exp. I. Cells of the FA + HT + CC group were firstly treated with an AMPK inhibitor (Compound C, 10 μM, 10 h) and then cultured in FA medium with 50 μM HT for 24 h.

After the culture, cells were harvested by use of the trypsin digestion method. There were three replicates for all the tests.

#### 2.2.2. Mitochondrial Function

To assess mitochondrial function, the activities of citrate synthase (CS), and succinate dehydrogenase (SDH) were determined in cell lysate using commercial kits (Nanjing JianCheng Bioengineering Institute, Nanjing, China). The ATP content was measured by chemiluminescence using a commercial assay kit (Beyotime, Shanghai, China). A JC-1 fluorescent probe (Beyotime, Shanghai, China) was used in mitochondrial membrane potential (MMP) measurement, as described in the manufacturer’s instructions. The fluorescence was detected by a flow cytometer (CytoFLEX, Beckman Coulter, Miami, FL, USA).

#### 2.2.3. Fluorescence Image

To observe fat droplets in ZFL, the BODIPY staining was conducted as described in our previous study [25]. To assess the mitophagy state of ZFL, cells were plated on μ-slide 8 well (ibidi, Gräfelfing, Germany). Before treatment, ZFL was incubated in 100 nM Mtphagy Dye (Dojindo, Japan) and washed twice with the medium without FBS. Then, 5 μg/mL Hoechst 33342 (Invitrogen, Carlsbad, CA, USA) was used to stain the nuclei for 10 min at room temperature. To assess the co-localization of mitochondria and lysosomes, cells were stained by 300 nM Mitotracker Red (Invitrogen, USA) and 75 nM Lyso-Tracker Green (Beyotime, Shanghai, China) for 20 min, respectively. Fluorescence imaging was performed and photographed using a Leica sp8 laser scanning confocal microscope.

### 2.3. Gene Expression

Total RNA was isolated by a reagents kit (RC101-01, Vazyme Biotech Co., Ltd., Nanjing, China) according to the manufacturer’s protocol. The RNA samples were treated with DNase to remove residual DNA, and the RNA purity and integrity were determined according to our previous study [23]. Complementary DNA (cDNA) generated from 0.5 μg RNA using a reagents kit (R211-01, Vazyme Biotech Co., Ltd., Nanjing, China) according to the manufacturer’s protocol.

cDNA samples were used to determine mRNA levels by Quantitative Real-time PCR (qRT-PCR), according to our previous study [23]. The expressions of genes were calculated by 2^−∆∆Ct^ method. Primers used in this study are shown in Table S2.

### 2.4. Western Blot

Western blots were conducted as described in our previous study [26]. The primary antibodies used in the present study included GAPDH (ab181602, Abcam, Cambridge, MA, USA), PINK1 (10006283, Cayman Chemical, Ann Arbor, MI, USA), LC3B (ab48394, Abcam, Cambridge, MA, USA), and phospho-AMPKα (#2535, Cell Signaling Technology, Danvers, MA, USA).

### 2.5. Statistical Analysis

Data were analyzed by one-way ANOVA, using SPSS 20 program. The multiple comparisons between experimental groups were compared by Tukey’s test. The level of significance was set at *p* < 0.05. All data are presented as means ± SE., and values with different uppercase letters are significantly different.

## 3. Results

### 3.1. In Vivo Study Using Spotted Seabass

#### 3.1.1. Growth, Fat Deposition and Blood Biochemistry

**Figure 1:**
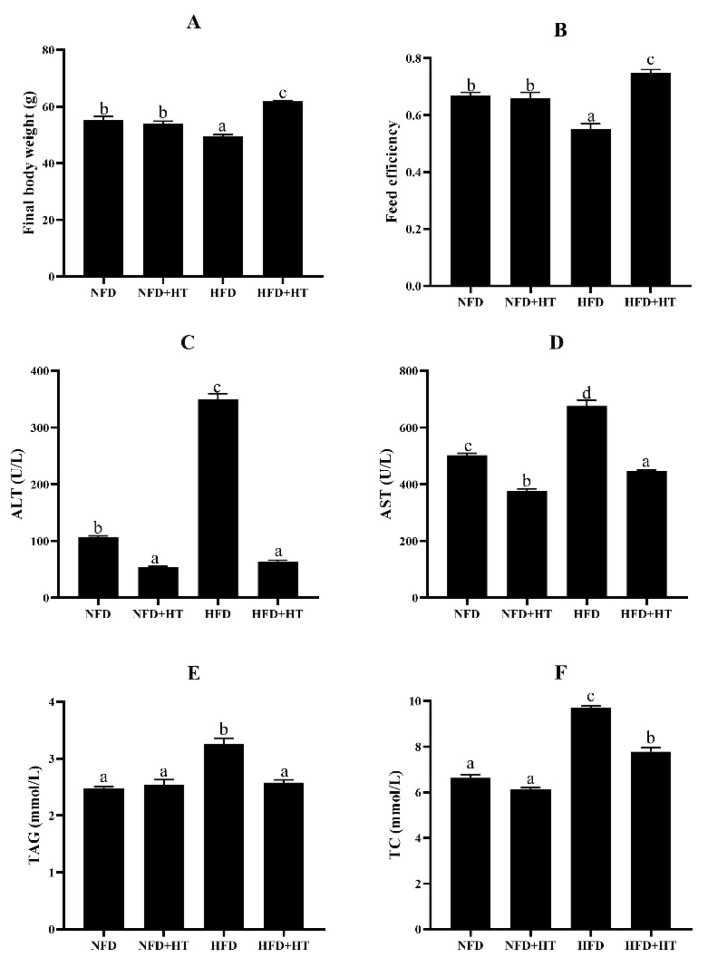
Final body weight (**A**), feed efficiency (**B**), plasma aspartate aminotransferase activity (AST: (**C**)), alanine aminotransferase activity (ALT: (**D**)), plasma triacylglycerol level (TAG: (**E**)), and total cholesterol (TC: (**F**)) content in spotted seabass (*L. maculatus*) fed the test diets for 8 weeks. All values are exhibited as mean ± SE. The values with different superscripts (a, b, c, d) are significantly different at *p* < 0.05 (Tukey’s test). Feed efficiency = wet weight gain/dry feed fed.

The HFD group exhibited dramatically lower final body weight (FBW) and feed efficiency (FE) than the other groups (*p* < 0.05). The addition of HT in HFD significantly improved FBW and FE (Figure 1A,B).

Significantly higher plasma AST and ALT activities were detected in the HFD group compared to other groups, and HT supplementation markedly decreased their activities (Figure 1C,D).

The HFD group showed significantly higher plasma TAG and TC concentrations, and HT remarkably reduced their concentrations (Figure 1E,F). Feeding HFD resulted in obvious enhancements of the abdominal fat index, hepatic TAG and TC levels, and HT addition ameliorated this phenomenon (Table 1).

#### 3.1.2. Histology of Liver

**Figure 2:**
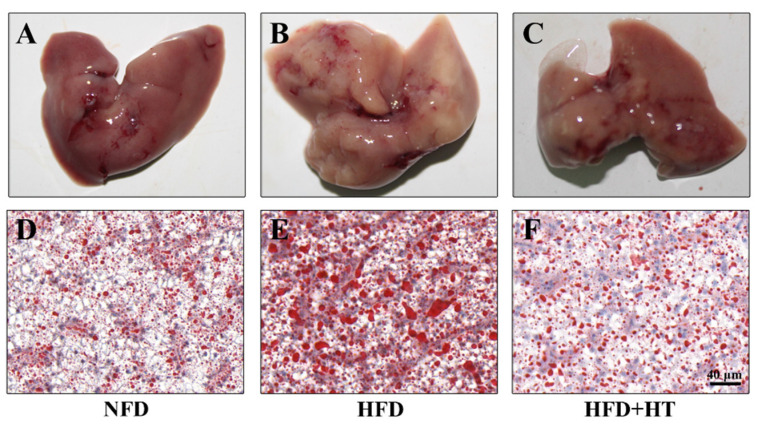
Histological examination of liver in spotted seabass (*L. maculatus*) fed the test diets for 8 weeks. Representative photographs (**A**–**C**) and oil red O-stained sections (**D**–**F**, scale bar = 20 μm).

The appearance of the liver in the HFD-fed fish exhibited a paler color, while the livers of fish in other groups exhibited a bright red color (Figure 2A–C). Meanwhile, Oil red O-staining showed more fat accumulation in the HFD group compared to the other groups, which confirms the fat-lowering effect of HT (Figure 2D–F).

#### 3.1.3. Oxidative Stress

**Figure 3:**
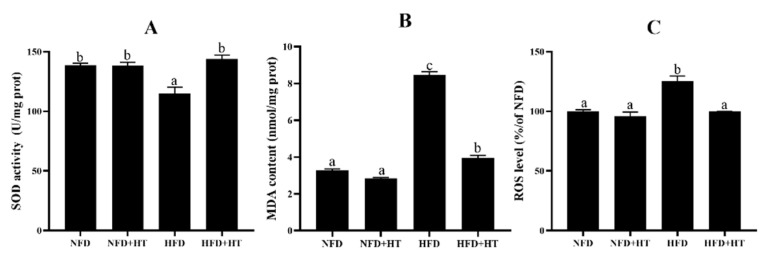
Superoxide dismutase activity (SOD: (**A**)), malondialdehyde content (MDA: (**B**)) and reactive oxygen species content (ROS: (**C**)) in the liver of *L. maculatus* fed the test diets for 8 weeks. All values are exhibited as mean ± SE. The values with different superscripts (a, b, c) are significantly different at *p* < 0.05 (Tukey’s test).

Noticeable reduction of SOD activity and enhancement of ROS and MDA concentrations were found in the HFD-fed fish. HT application enhanced SOD activity and lowered ROS and MDA levels (Figure 3A–C).

#### 3.1.4. Mitochondria

**Figure 4:**
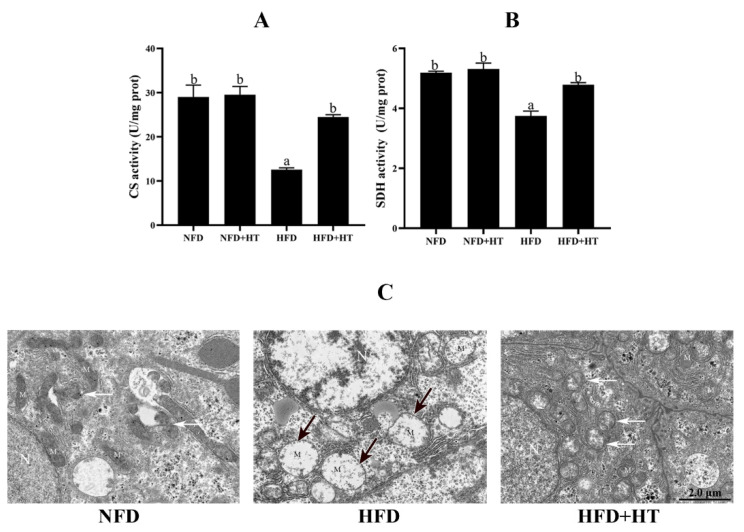
Citrate synthase (CS: (**A**)) and succinate dehydrogenase (SDH: (**B**)) activities and (**C**) transmission electron microscopy images of mitochondria (N—nucleus; M—mitochondrion; black arrows—damaged mitochondria; white arrows—mitochondrial autophagosomes) in the liver of *L. maculatus* fed the test diets for 8 weeks. All values are exhibited as mean ± SE. The values with different superscripts (a, b) are significantly different at *p* < 0.05 (Tukey’s test).

The activities of CS and SDH in the HFD group were significantly lower than other groups, while HT administration increased these activities (Figure 4A,B).

Transmission electron microscopy (TEM) images of hepatocytes displayed a normal mitochondrial ultrastructure in the NFD group. Hydropic changes and severe loss of cristae and matrix were observed in the mitochondria of the hepatocytes of HFD group. Intriguingly, the HFD + HT group showed healthy mitochondrial ultrastructure and more mitochondrial autophagosomes (Figure 4C). The analysis of TEM images showed that the amount of damaged mitochondia in the HFD group was much higher than that of two other groups (Figure S1)

**Figure 5:**
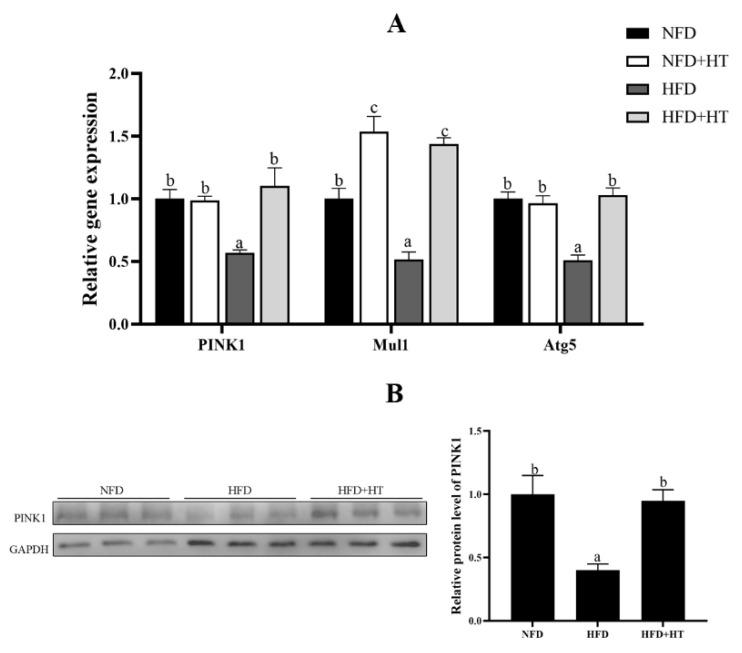
Relative gene expression of mitophagy-related genes (**A**) and protein level of PINK1 (**B**) in the liver of *L. maculatus* fed the test diets for 8 weeks. All values are exhibited as mean ± SE. The values with different superscripts (a, b, c) are significantly different at *p* < 0.05 (Tukey’s test).

Furthermore, expressions of mitophagy-related genes such as *PINK1*, *Mul1* and *Atg5* were markedly down-regulated by HFD. Addition of HT to HFD significantly can up-regulate these expressions (Figure 5A). Similarly, the protein level of PINK1 was significantly decreased in the HFD group, which was significantly improved by the addition of HT (Figure 5B).

### 3.2. In Vitro Study Using ZFL Cells

#### 3.2.1. Exp. I: Effects of HT on Fat Deposition and Mitochondria Function

**Figure 6:**
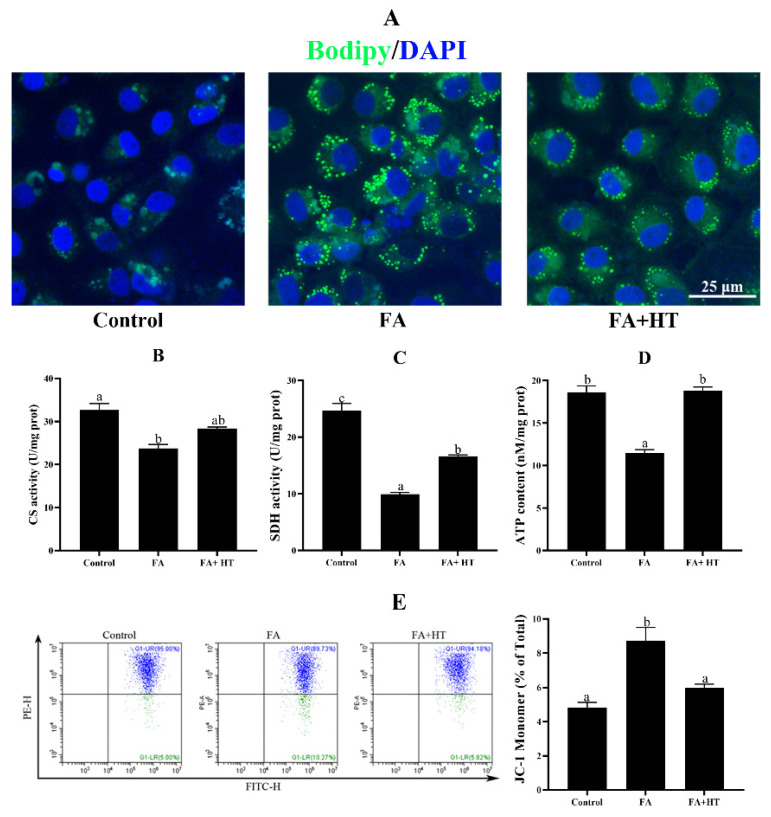
Staining of fat droplet by BODIPY 493/503 (green) in ZFL cell (**A**). Nuclei are highlighted with Hoechst 33342 (blue), scale bar = 25 μm. Citrate synthase (CS: (**B**)) and succinate dehydrogenase (SDH: (**C**)) activities and ATP content (ATP: (**D**)) in ZFL cell. Mitochondrial membrane potential (MMP) of ZFL cell was detected by the flow cytometer assessment of JC-1 staining (**E**). The ratio of JC-1 monomer is shown. Control group—complete LDF medium; FA group—FA medium (complete LDF medium with 0.25 mM oleic acid and 0.25 mM palmitic acid); FA + HT group—FA medium containing 50 μM of HT. All values are exhibited as mean ± SE. The values with different superscripts (a, b, c) are significantly different at *p* < 0.05 (Tukey’s test).

ZFL cells treated with 0.5 mM FA exhibited significantly higher TAG content compared to the control group, and HT addition decreased its concentration (Figure S2). To further confirm the lipid-lowering effect of HT, BODIPY 493/503 was used to stain the lipid droplets (Figure 6A). The FA group showed a significantly higher number of green dots than the other groups which is in tune with TAG content.

Activities of citrate synthase (CS) and succinate dehydrogenase (SDH), and the level of ATP in ZFL cells significantly decreased in the FA group. HT addition significantly enhanced the ATP content and SDH activity. CS activity was also increased by HT application but the difference was not significant (Figure 6B–D). Furthermore, the mitochondrial membrane potential (MMP) was significantly dysregulated in the ZFL cells treated with 0.5 mM FA which was significantly reversed by HT application (Figure 6E).

**Figure 7:**
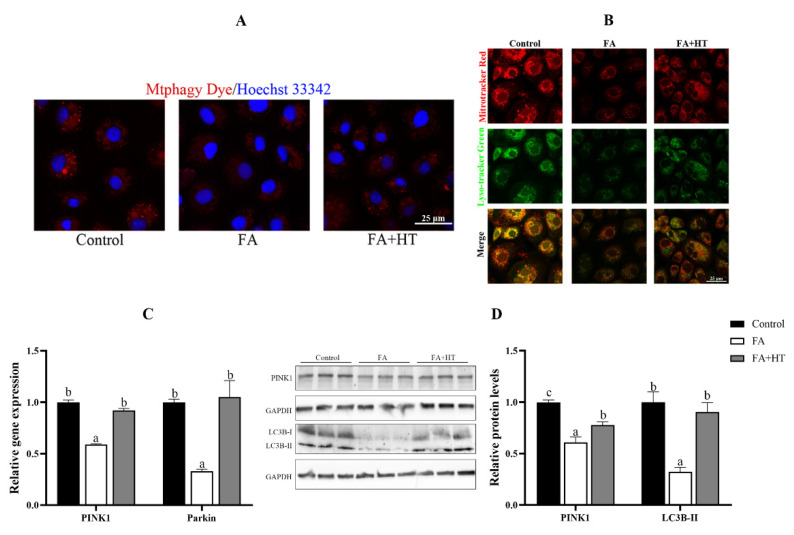
Staining of mitochondrial autophagosomes by Mtphagy Dye (red) in ZFL cell (**A**). Nuclei are highlighted with Hoechst 33342 (blue). The co-localization of Mitochondrion (Red) and Lysosome (Green) in ZFL cell (yellow dots), (**B**). Scale bar = 25 μm. The relative gene expression level of *PINK1* and *Parkin* (**C**). The relative level of PINK1 and LC3B-II proteins in ZFL cells (**D**). All values are exhibited as mean ± SE. The values with different superscripts (a, b, c) are significantly different at *p* < 0.05 (Tukey’s test).

Mtphagy Dye is a novel fluorescent small molecule with good sensitivity and photostability for detecting mitophagy phenomena and is often regarded as a reliable tool to indicate mitochondrial autophagy. The results showed the significant reduction of red dots in FA group compared to the control and FA + HT groups (Figure 7A). The classical mitophagy pathway relies on lysosomes for the removal of superfluous or damaged mitochondria. Hence, co-localization of mitochondria via lysosomes is used as a known indicator of mitophagy. The result revealed drastically lower co-localization of mitochondria with lysosomes (yellow dots) in FA group than the control and FA + HT groups (Figure 7B). Furthermore, the gene expression level of *PINK1* and *Parkin* was down-regulated by FA, while HT significantly improved the values (Figure 7C). Western blot analysis indicated the reduction of PINK1 and LC3B II protein levels in the FA group, and HT supplementation significantly enhanced their abundance (*p* < 0.05) (Figure 7D).

#### 3.2.2. Exp. II: Mitophagy Inhibitor Csa Blocked HT-Mediated Effects

We used cyclosporin A (Csa) as an inhibitor for inhibiting the degree of mitophagy. The Mtphagy Dye straining showed the suppression of mitophagy by Csa treatment, as the FA + HT + Csa group exhibited fewer red dots than the other groups (Figure S3).

**Figure 8:**
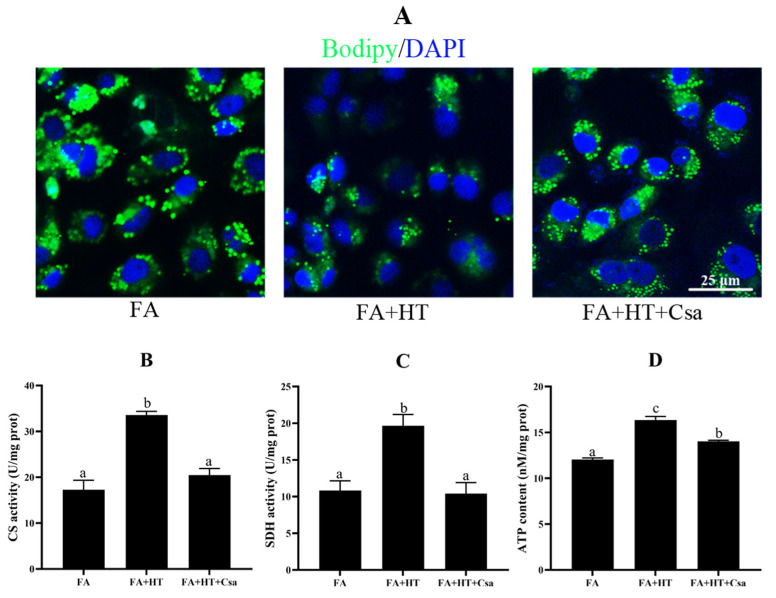
Staining of fat droplet by BODIPY 493/503 (green) in ZFL cell (**A**). Nuclei are highlighted with Hoechst 33342 (blue), scale bar = 25 μm (**A**). Citrate synthase (**B**) and succinate dehydrogenase (CS: (**C**)) activities and ATP content (SDH: (**D**)) in ZFL cell. Values are shown as mean ± SE (n = 3). FA group—FA medium; FA + HT group—FA medium containing 50 μM HT; FA + HT + Csa group—FA medium containing 50 μM HT with Csa pretreatment. All values are exhibited as mean ± SE. The values with different superscripts (a, b, c) are significantly different at *p* < 0.05 (Tukey’s test).

The BODIPY staining showed a significantly higher number of green dots in FA + HT + Csa group compared to the FA + HT group (Figure 8A), and the TAG content was also increased by Csa (Figure S4) indicating that Csa treatment partially blocked the HT-mediated fat-lowering effect. Moreover, the Csa application dramatically abrogated the HT-induced enhancements of ATP content, and CS and SDH activities (*p* < 0.05) (Figure 8B–D).

#### 3.2.3. Exp. III: Compound C Down-Regulated the Expression of AMPK/PINK1 Pathway

**Figure 9:**
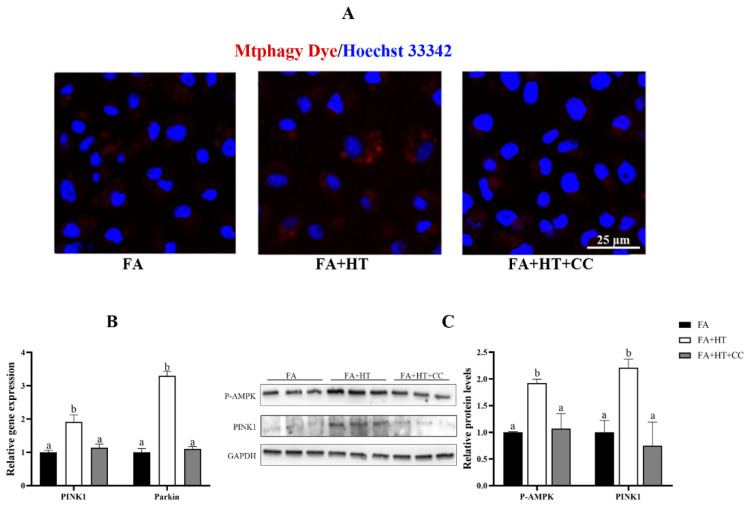
Staining of mitochondrial autophagosomes by Mtphagy Dye (red) in ZFL cell (**A**). Nuclei are highlighted with Hoechst 33342 (blue), scale bar = 25 μm (**A**). The relative gene expression level of *PINK1* and *Parkin* (**B**). Western blot analysis of PINK1 and P-AMPK in ZFL cell (**C**). FA group—FA medium (complete LDF medium with 0.25 mM oleic acid and 0.25 mM palmitic acid); FA + HT group—FA medium containing 50 μM HT, FA + HT + Csa group—FA medium containing 50 μM HT with CC pretreatment. All values are exhibited as mean ± SE. The values of bar graphs with different superscripts (a, b) are significantly different at *p* < 0.05 (Tukey’s test).

**Figure 10:**
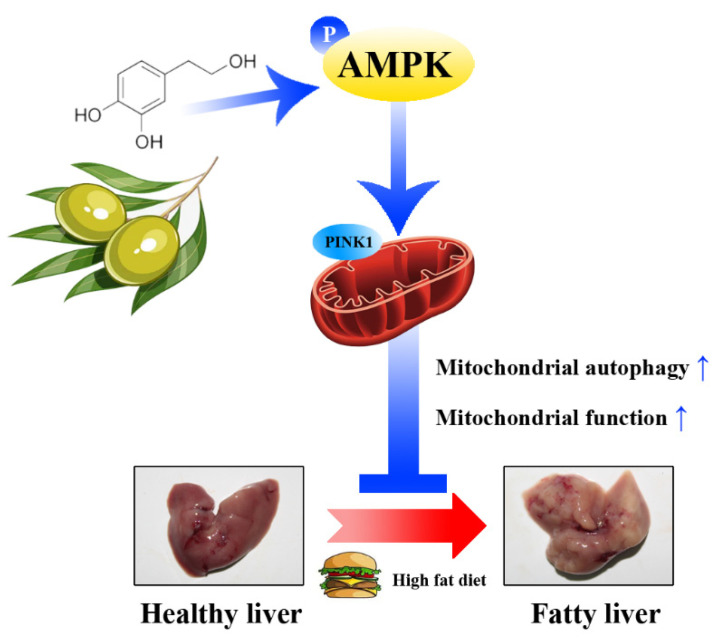
A schematic diagram showing the main internal mechanism of the lipid-lowing effect of HT. HT activates mitochondrial autophagy through the AMPK/PINK1 pathway, promotes mitochondrial function, which alleviates hepatic fat accumulation.

Compound C (CC) was used for inhibiting AMPK activity in this study. The Mtphagy Dye straining showed the suppression of mitophagy level by CC treatment identified by reduction of red dots in FA + HT + CC group (Figure 9A). Moreover, the gene expression of *PINK1* and *Parkin* was activated by HT administration, while CC treatment blocked their expression (Figure 9B). Moreover, HT treatment significantly enhanced PINK1 and P-AMPK protein levels (Figure 10), which were evaded by CC addition (*p* < 0.05) (Figure 9C).

## 4. Discussion

Fats can yield double the energy of carbohydrates/proteins because of their high-energy bonds. Thus, storing energy as fat is ubiquitous for all organisms, from simple prokaryotes to humans [17,18]. The central players of fat storage in lower organisms are similar to those in higher organisms [21]. Therefore, studying the regulation of fat storage in fish can contribute to our understanding of human metabolic disorders. Moreover, several characteristics of spotted seabass such as convenience, low cost [19], and reflecting the degree of hepatic fat deposition by changes in the color of liver make it a suitable fish model.

The same as mammals, intake of HFD results in excessive fat deposition and hepatic damage in fish [20]. Our results showed the increase of liver TAG content and activity of plasma transaminases in fish fed HFD, indicating liver damage. HT attenuated the HFD-induced excessive fat deposition in both the liver and abdomen, indicating its fat-lowering effects as evidenced in rodents [27]. It has been reported by several authors that fat accumulation in tissues can enhance lipid oxidation rate leading to oxidative stress [28]. Likewise, the results of present study showed that feeding HFD leads to increased ROS and MDA levels, and HT supplementation mitigated the HFD-induced liver oxidative stress. This could be associated with ortho dihydroxylphenol group of HT which exerts a great ROS-scavenging capacity [29]. Furthermore, the transcriptional factor Nrf2 has been confirmed as a key regulation point of the antioxidant enzymes system, some previous studies indicated that HT-enhanced activities and abundances of antioxidative enzymes through the Nrf2/Keap1 pathway activation, which improved antioxidant enzymes activity and mitochondrial oxidative status [30,31].

In response to excessive fat intake, mitochondria can induce FA oxidation rate and supply reducing equivalents for the mitochondrial electron transport chain, which may trigger increased ROS formation [32]. The enhanced production of ROS often induces mitochondrial damage [7]. Structural and molecular alterations of mitochondria, including the loss of cristae and impaired chain enzymes activity, are often observed during fat overload [12,22,23]. Results of our in vivo study indicated that HT supplementation can attenuate fat deposition, oxidative stress and the mitochondrial damages caused by HFD intake. Considering the key roles of mitochondria in these processes, we assume that HT acts through its regulation on mitochondrion. To explore the molecular mechanism, the ZFL was used as a model for our in vitro studies.

Fat droplets in cells can be labelled by BODIPY to determine the degree of fat deposition [33]. Our in vitro test using ZFL indicated that HT treatment significantly reduces fat deposition in cells. It has been shown that mitochondrial abnormalities are often accompanied with impaired β-oxidation and excessive fat deposition [22,25]. Citrate synthase (CS) and succinate dehydrogenase (SDH) are key points of the tricarboxylic acid cycle, and their activities are regarded as indicators of mitochondrial function [34]. Enhancement of CS and SDH activities in the HT-treated group may indicate the improvement of mitochondrial function. Furthermore, mitochondrial dysfunction induces respiratory chain injury and a lack of ATP [35]. Our data indicated that FA-induced lack of ATP was reversed by HT application, further confirming the promotion effects of HT on mitochondrial function. Mitochondrial membrane potential (ΔΨm) as a key maker of mitochondrial function decreases when mitochondria are damaged [36]. Alterations of ΔΨm in cultured cells were evaluated in this study and the results showed that FA treatment decreases ΔΨm, which was subsequently attenuated by HT application, confirming the beneficial effects of HT on mitochondrion.

As the highly dynamic organelles, the mitochondria act the key roles in lipid metabolism and cellular homeostasis. Cells require a wide arsenal of quality control mechanisms to prevent mitochondrial damage. Mitochondrial biogenesis and mitophagy are two pivotal processes that maintain mitochondrial homeostasis [37]. Under normal conditions, damaged or superfluous mitochondria are cleared mainly through specific autophagy-lysosome pathways called mitophagy. The impairment of autophagy–lysosome signaling causes the formation of excess ROS, mitochondrial dysfunction and the activation of programmed cell death [38]. Considering the observed mitochondrial autophagosomes, we assume that HFD-induced oxidative stress and mitochondrial dysfunction might be associated with the impairment of autophagic machinery. Thus, a fluorescent small molecule named Mtphagy Dye was used in this study to determine the degree of mitophagy. The results showed the suppression of the level of mitophagy by FA treatment was improved by HT application. It has been acknowledged that there are various forms of mitochondrial autophagy including microautophagy, cytosol-to-vacuole transport, and macroautophagy [39]. The process of macroautophagy consists of formation of isolation membranes, enclosing various intracellular components inside, and formation of double-membrane vesicles called autophagosomes [40]. Subsequently, the autophagosomes fuse with lysosomes to generate autolysosomes allowing the degradation of the autophagosomal contents [41]. Hence, to confirm the results we studied the co-localization of mitochondria and lysosomes using the laser scanning confocal microscope. Expectedly, the FA group showed lesser co-localization of mitochondria with lysosomes, while more co-localization was found for the HT group. This result confirmed the suppression of the level of mitophagy by FA treatment and its improvement with HT application. Moreover, our findings revealed the similarity of the mitophagy process in fish and humans, as macroautophagy is the main type of mitophagy in humans.

The molecular mechanisms underlying mitochondrial autophagy have been characterized recently [42]. It has been reported that mitochondrial membrane-anchored protein kinase PINK1 plays a central role during the autophagic process [43]. After the loss of mitochondrial membrane potential (ΔΨm), PINK1 is stabilized on the outer mitochondrial membrane and activates parkin ubiquitin ligase activity to form mitophagosome. Finally, mitophagosome is fused with lysosome for degradation of damaged mitochondria [44]. In the current study, the expression of PINK1 was down-regulated by FA treatment, and application of HT up-regulated its expression. It confirms that FA suppressed mitophagy, and HT application enhanced it through the PINK1 pathway.

According to the results, we postulated that HT regulates mitochondrial function via promoting degradation of the damaged mitochondria. Thus, we employed Csa as an inhibitor for inhibiting the degree of mitophagy. The results showed that the effects of HT on lowering fat and mitochondrial function disappears following Csa treatment. Recently, mitophagy has also been suggested as a prequisite for mitochondrial biogenesis [45]. This indicates that mitophagy acts not only via clearance of damaged mitochondria but also through the biogenesis of new mitochondria. Based on these findings, we deduce that HT enhances mitochondrial function through activating the autophagy process.

Research in mammals revealed the effects of AMP-activated protein kinase (AMPK) on fat metabolism [46,47]. Activation of AMPK induces down-regulation of anabolic pathways including de novo synthesis of triglyceride and cholesterol and, in the meantime, up-regulation of catabolic pathways such as lipolysis [48]. Interestingly, it has also been reported that AMPK can activate mitophagy via PINK1 phosphorylation in the heart failure model of mammals [49]. In the present study, we found that HT activated the expression of PINK1 and p-AMPK, which indicated that HT promotes mitophagy through the activation of AMPK/PINK1 pathway. Furthermore, Compound C was used in this study for inhibiting AMPK activity and the results showed that suppression of AMPK markedly counteracted the activation of mitophagy and PINK1 expression by HT. This finding suggests that mitophagy activating property of HT is mediated through the AMPK/PINK1 pathway.

## 5. Conclusions

To conclude, our findings demonstrated that HT alleviates fat accumulation, oxidative stress and mitochondrial dysfunction, and its effects are suggested to be relied on for activating mitophagy through the AMPK/PINK1 pathway.

## Figures and Tables

**Table 1:** Fat deposition in spotted seabass (*L. maculatus*) fed the test diets for 8 weeks.

	NFD	HFD	NFD + HT	HFD-HT
Abdominal fat index (%) ^1^	4.13 ± 0.09 ^a^	6.86 ± 0.20 ^c^	4.16 ± 0.09 ^a^	5.02 ± 0.08 ^b^
Liver TAG (nmol/g tissue)	4.33 ± 0.13 ^ab^	6.24 ± 0.17 ^c^	3.94 ± 0.20 ^a^	4.91 ± 0.07 ^b^
Liver TC (nmol/g tissue)	4.56 ± 0.38 ^a^	6.99 ± 0.07 ^c^	4.64 ± 0.19 ^a^	5.79 ± 0.13 ^b^

Values are shown as mean ± SE (n = 3). The values with different superscripts (a, b, c) are significantly different at *p* < 0.05 (Tukey’s test). NFD—normal-fat diet (10% fat); HFD—high-fat diet (16% fat); NFD + HT—NFD supplemented with 200 mg/kg of hydroxytyrosol (HT); HFD + HT—HFD supplemented with 200 mg/kg of HT. ^1^ Abdominal fat index (%) = 100 × abdominal fat/body weight.

## Data Availability

The data is contained within the article and Supplementary Materials.

## Supplementary Materials

The following supporting information can be downloaded at: https://www.mdpi.com/article/10.3390/antiox11050893/s1, Table S1: Formulation and Proximate Composition of Experimental Diets; Table S2: Sequences of Primers Used for RT-qPCR; Figure S1: The number of normal mitochondria (A) and damaged mitochondria (B) in the liver TEM images of spotted seabass (*L. maculatus*) fed the test diets for 8 weeks; Figure S2: TAG content of ZFL cells in control, FA and FA + HT groups; Figure S3: Mtphagy Dye of ZFL cell in FA, FA + HT and FA + HT + Csa groups. Figure S4: TAG content of ZFL cell in FA, FA + HT and FA + HT + Csa groups
