# Supplementary material for: Hydroxytyrosol Promotes the Mitochondrial Function through Activating Mitophagy
Source: Antioxidants (Basel). 2022 Apr 30;11(5):893. doi: 10.3390/antiox11050893 (PMC9138034; doi:10.3390/antiox11050893)
Supplement: Supplementary file 1 [file antioxidants-11-00893-s001.zip › antioxidants-1663154-supplementary.pdf]

Table S1. Formulation and proximate composition of experimental diets.

Ingredients (g/kg)	NFD	HFD	NFD+HT	HFD+HT
Fish meal	268.5	268.5	268.5	268.5
Poultry meal	84.7	84.7	84.7	84.7
Gluten flour	73	87.5	73	87.5
Soybean meal	250	250	250	250
α-starch	30	30	30	30
Wheat Flour	201.1	125.1	200.9	124.9
Fish oil	19.3	50	19.3	50
Soybean oil	19.3	50	19.3	50
Soybean lecithin	20	20	20	20
Premix[1]	13.5	13.5	13.5	13.5
Calcium biphosphate	20.8	20.8	20.8	20.8
Hydroxytyrosol	-	-	0.2	0.2
**Proximate composition (g/kg)**				
Protein	440	440	440	440
Lipid	100	160	100	160

[1]Premix supplied the following minerals (g/kg) and vitamins (IU or mg/kg): CuSO4·5H2O, 2.0 g; $FeSO_4·7H_2O$, 25 g; $ZnSO_4·7H_2O$, 22 g; $MnSO_4$·4H2O, 7 g; Na_2SeO_3, 0.04 g; KI, 0.026 g; $CoCl_2·6H_2O$, 0.1 g; Vitamin A, 900000 IU; Vitamin D, 200000 IU; Vitamin E, 4500 mg; Vitamin K3, 220 mg; Vitamin B1, 320 mg; Vitamin B2, 1090 mg; Niacin, 2800 mg; Vitamin B5, 2000 mg; Vitamin B6, 500 mg; Vitamin B12, 1.6 mg; Vitamin C, 5000 mg; Pantothenate, 1000 mg; Folic acid, 165 mg; Choline, 60000 mg.

Table S2. Sequences of primers used for RT-qPCR.

Target gene	Forward (5'-3')	Reverse (5'-3')
PINK1[1]	CTGTGAAAGCCCGGTACACT	TGATGTGGAACTTTGGGGCA
Muil1[1]	GCTGCCGTGATACGAGTCAT	ACGTTGGACAAGGACTGGAC
Atg5[1]	TCAGTCGCTGCCATTAGAGC	TCTCGTCACCTGCGAAAACT
β-actin[1,a]	TCGAGCACGGTATTGTGACC	TCAGGTGCAACTCTCAGCTC
PINK1[2]	TCGGGAAAGGTTGTAACGCA	TTCGGGTTCAGTTCCACCAA
Parkin[2]	GGCTGTCCTGATTCGCTCAT	TGAAGAACACACTCCTCCGC
β-actin[2,a]	GCCATGGATGAGGAAATCGC	TCTGGGTCGTCCAACAATGG

[1]Primers for spotted seabass

[2]Primers for zebrafish

[a]Reference gene

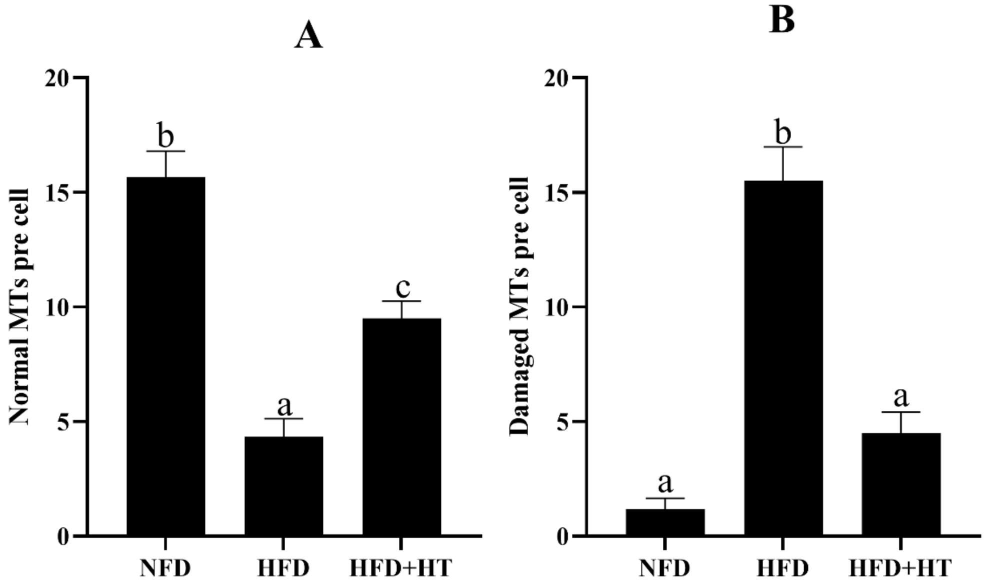

Figure S1. The number of normal mitochondria (A) and damaged mitochondria (B) in the liver TEM images of spotted seabass (*L. maculatus*) fed the test diets for 8 weeks. Values are shown as mean ± SE (n=6). Different letters indicate significant difference differences among groups ($P < 0.05$). NFD: normal fat diet (10% fat), HFD: high fat diet (16% fat), NFD+HT: NFD supplemented with 200 mg/kg of hydroxytyrosol (HT), HFD+HT: HFD supplemented with 200 mg/kg of HT.

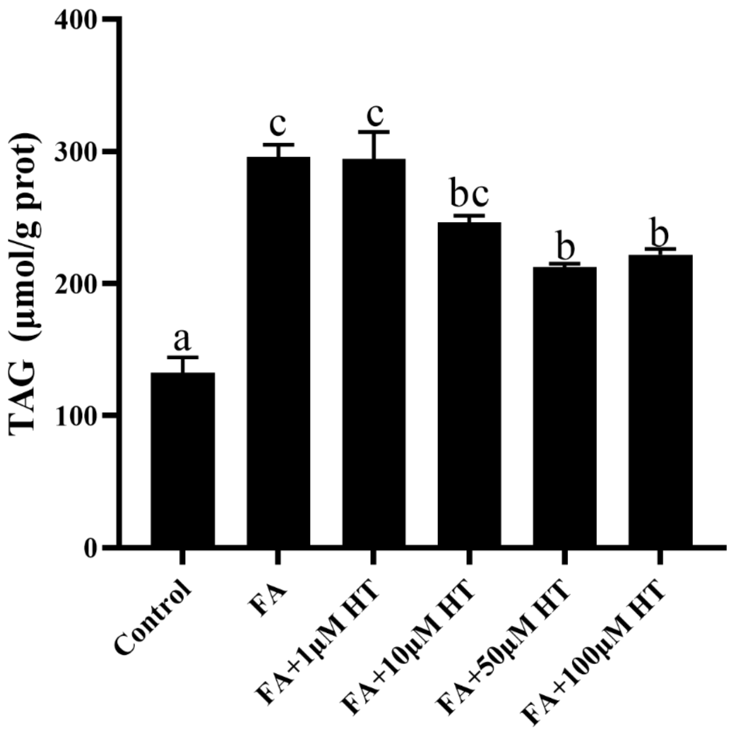

Figure S2. TAG content in ZFL cells (mean ± SE, n=3). Control group: LDF medium, FA group: FA medium (LDF medium with 0.25 mM oleic acid and 0.25 mM palmitic acid), FA+HT group: FA medium adding different HT concentration. All values are exhibited as mean ± SE. Different letters show significant differences ($P < 0.05$).

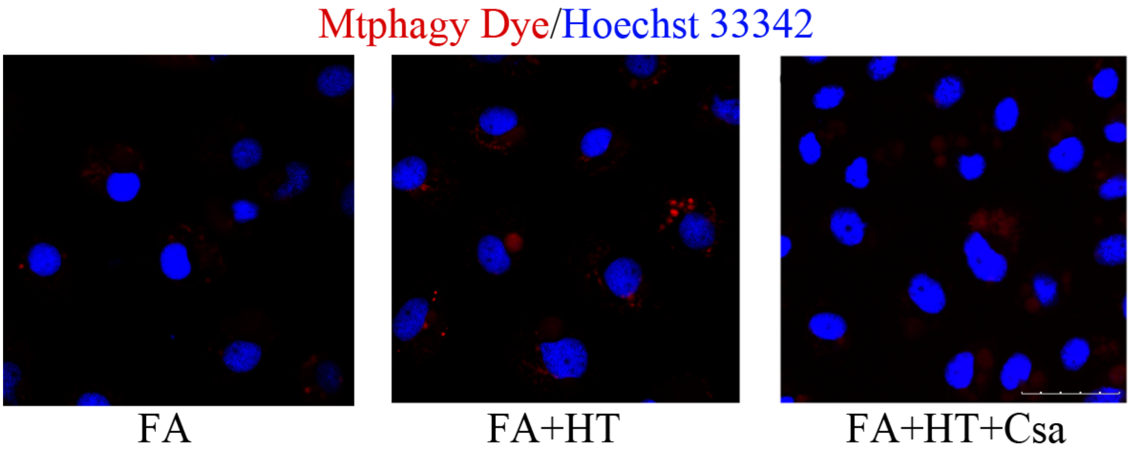

Figure S3. Staining of the mitochondrial autophagosome by Mtphagy Dye (red) in ZFL cell line. Nuclei are highlighted with Hoechst 33342. Scale bar = 25 μm

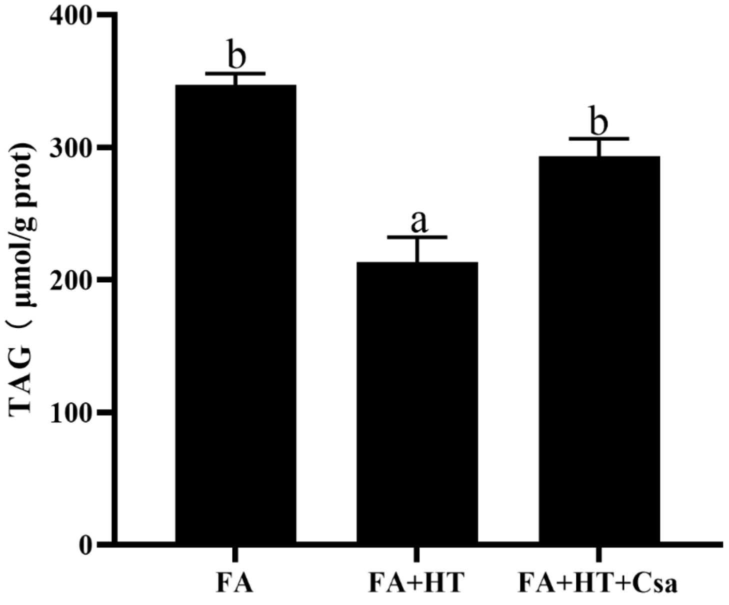

Figure S4. TAG content in ZFL cell line. All values are exhibited as mean ± SE. Different letters show significant differences ($P < 0.05$).
